# ACTP: A webserver for predicting potential targets and relevant pathways of autophagy-modulating compounds

**DOI:** 10.18632/oncotarget.7015

**Published:** 2016-01-25

**Authors:** Tao Xie, Lan Zhang, Shouyue Zhang, Liang Ouyang, Haoyang Cai, Bo Liu

**Affiliations:** ^1^ State Key Laboratory of Biotherapy and Cancer Center, West China Hospital, Sichuan University, and Collaborative Innovation Center of Biotherapy, Chengdu 610041, China; ^2^ Center of Growth, Metabolism, and Aging, Key Laboratory of Bio-Resources and Eco-Environment, College of Life Sciences, Sichuan University, Chengdu 610064, China

**Keywords:** autophagic compound-target prediction (ACTP), autophagy (macroautophagy), autophagy-activating compound, autophagy-inhibiting compound, webserver

## Abstract

Autophagy (macroautophagy) is well known as an evolutionarily conserved lysosomal degradation process for long-lived proteins and damaged organelles. Recently, accumulating evidence has revealed a series of small-molecule compounds that may activate or inhibit autophagy for therapeutic potential on human diseases. However, targeting autophagy for drug discovery still remains in its infancy. In this study, we developed a webserver called Autophagic Compound-Target Prediction (ACTP) (http://actp.liu-lab.com/) that could predict autophagic targets and relevant pathways for a given compound. The flexible docking of submitted small-molecule compound (s) to potential autophagic targets could be performed by backend reverse docking. The webpage would return structure-based scores and relevant pathways for each predicted target. Thus, these results provide a basis for the rapid prediction of potential targets/pathways of possible autophagy-activating or autophagy-inhibiting compounds without labor-intensive experiments. Moreover, ACTP will be helpful to shed light on identifying more novel autophagy-activating or autophagy-inhibiting compounds for future therapeutic implications.

## INTRODUCTION

Autophagy refers to an evolutionarily conserved, multi-step lysosomal degradation process, in which a cell degrades long-lived proteins and damaged organelles [1, 2]. Macroautophagy (hereafter referred to as autophagy) is a major, regulated catabolic mechanism that involves the delivery of cytoplasmic cargo sequestered inside double-membrane vesicles to the lysosome [3], and is linked to several pathological processes, including cancers and neurodegenerative diseases [4, 5]. Autophagy is considered as a physiological mechanism that may serve as a means for temporary survival and provide a way to recycle macromolecules as an alternative energy source. If cellular stress leads to continuous or excessively induced autophagy, cell death will ensue [6, 7]. Several studies have reconciled the opposing roles of autophagy in diseases and demonstrated that autophagy can act as either a guardian or executioner [8–11]. The different roles of autophagy depend on disease stages, surrounding cellular environment, and attempted therapeutic interventions [12–15].

Accordingly, targeting autophagy may be a promising therapeutic strategy for treatment of diseases. Recently, accumulating evidence has revealed many small-molecule compounds that can activate or inhibit autophagy and may therefore have remarkable therapeutic potential on diseases, such as cancers and neurodegenerative diseases [16, 17]. However, targeting autophagy for drug development remains in its infancy. Here, we designed a webserver called Autophagic Compound-Target Prediction (ACTP) (http://actp.liu-lab.com/) that can predict potential autophagic targets and relevant pathways for given compound (s). The ACTP webserver will help to explore more possible autophagy-activating or autophagy-inhibiting drugs for potential therapeutic purposes.

## RESULTS

### Potential autophagic targets in ACTP

**Figure 1:**
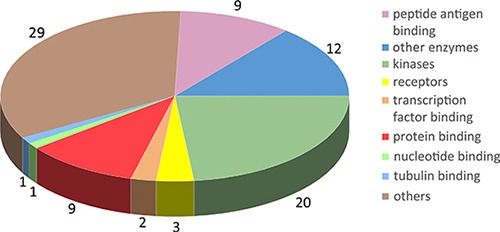
Molecular classification of potential autophagic targets Herein, 86 targets with crystal structures were assigned to the following groups: peptide antigen binding, other enzymes, kinases, receptors, transcription factor binding, protein binding, nucleotide binding, tubulin binding and others. Groups are marked with different colors. The number of targets contained in each group is displayed in the pie chart.

We collected 430 target proteins (199 were reviewed, and 231 were unreviewed) from Uniprot. The basic protein information included the accession number, full name, and molecular function. We identified GO annotation terms and related diseases information from the Online Mendelian Inheritance in Man (OMIM) database. Crystal structures of 86 targets were downloaded from the Protein Data Bank (PDB) and saved as 948 PDB files. Six hundred and fifteen PDB structures were selected as available structures for docking, and their PDB codes were also saved (Table 1 and Supplementary Table S1). We prefer to retain PDBs that have both high resolution and complete amino acid motif covering active sites and compound-binding sites. For those PDBs have better resolution and worst coverage than a second one, we will firstly consider the sequence integrity (that means the PDB entry has a complete amino acid motif covering active sites and compound-binding sites) rather than resolution; thus, we will retain PDBs have complete amino acids motif even if they have relative lower resolution. For those PDBs have lower resolution and worst coverage, we will perform homology modeling instead of using these PDBs. These proteins were assigned to the following 9 functional target groups: antigen, enzyme, kinase, receptor, protein binding, nucleotide binding, transcription factor binding, tubulin binding, and others (Figure 1). For reviewed proteins without available crystal structures and the BLAST result with the template shown > 30% similarity, we performed homology modeling to generate predicted structures using Discovery Studio 3.5 (Supplementary Table S2 and Supplementary Table S3). 109 protein sequence files were downloaded from Uniprot and saved in FASTA format. Then, the templates were found using BLAST. Finally, the structures of 109 targets were generated and saved in PDB format. In addition, the PDB files were available from the corresponding PDB number hyperlink on the result page of the webserver. For example, the mTOR file contains the following information: the accession number, “P42345”; the name, “Serine/threonine-protein kinase mTOR (Mechanistic target of rapamycin)”; and the function, “Serine/threonine protein kinase is a central regulator of cellular metabolism, growth and survival in response to hormones, growth factors, nutrients, energy, and stress signals. mTOR can activate or inhibit the phosphorylation of at least 800 proteins directly or indirectly.” The PDB accession number for mTOR is 4dri, and the PDB file was downloaded from http://rcsb.org. Discovery Studio 3.5 was then used to prepare the PDB file for docking by deleting water, cleaning the protein, and detecting the interaction site.

**Table 1:** Structure-based autophagic targets (Reviewed)

Uniprot ID	Uniprot Accession	Number of PDBs	Target type	Uniprot ID	Uniprot Accession	Number of PDBs	Target type
P53_HUMAN	P04637	74	antigen	5HT2B_HUMAN	P41595	2	receptor
DRA_HUMAN	P01903	55	antigen	NR1D1_HUMAN	P20393	5	receptor
2B11_HUMAN	P04229	34	antigen	B2CL1_HUMAN	Q07817	38	protein binding
2B14_HUMAN	P13760	9	antigen	RGS19_HUMAN	P49795	1	others
DRB5_HUMAN	Q30154	4	antigen	BAD_HUMAN	Q92934	1	protein binding
ITB4_HUMAN	P16144	8	antigen	BCL2_HUMAN	P10415	11	protein binding
DRB3_HUMAN	P79483	3	antigen	TAU_HUMAN	P10636	6	protein binding
DPB1_HUMAN	P04440	3	antigen	S100A9_HUMAN	P06702	3	others
DPA1_HUMAN	P20036	3	antigen	PARK7_HUMAN	Q99497	26	others
PRKN2_HUMAN	O60260	4	enzyme	S100A8_HUMAN	P05109	3	others
SIR2_HUMAN	Q8IXJ6	5	enzyme	SQSTM_HUMAN	Q13501	5	protein binding
CATD_HUMAN	P07339	4	enzyme	MLP3B_HUMAN	Q9GZQ8	6	others
ATG4B_HUMAN	Q9Y4P1	4	enzyme	PA1B2_HUMAN	P68402	1	others
ATG4A_HUMAN	Q8WYN0	2	enzyme	NBR1_HUMAN	Q14596	8	others
UBP13_HUMAN	Q92995	2	enzyme	GOPC_HUMAN	Q9HD26	8	others
HDAC6_HUMAN	Q9UBN7	3	enzyme	SPT5H_HUMAN	O00267	5	protein binding
TIGAR_HUMAN	Q9NQ88	1	enzyme	MT3_HUMAN	P25713	2	others
SIR1_HUMAN	Q96EB6	4	enzyme	RAB1A_HUMAN	P62820	8	nucleotide binding
ATG3_HUMAN	Q9NT62	1	enzyme	BNIP3_HUMAN	Q12983	2	protein binding
HERC1_HUMAN	Q15751	2	enzyme	PSN1_HUMAN	P49768	1	protein binding
EPM2A_HUMAN	O95278	2	enzyme	GBRL1_HUMAN	Q9H0R8	2	tubulin binding
MK14_HUMAN	Q16539	103	kinase	GATA4_HUMAN	P43694	1	transcription factor binding
AKT1_HUMAN	P31749	13	kinase	IF16_HUMAN	Q16666	4	others
CDK5_HUMAN	Q00535	5	kinase	BECN1_HUMAN	Q14457	5	others
DAPK1_HUMAN	P53355	21	kinase	TBC14_HUMAN	Q9P2M4	1	others
KPYM_HUMAN	P14618	16	kinase	FOXO1_HUMAN	Q12778	2	others
MK08_HUMAN	P45983	20	kinase	MLP3A_HUMAN	Q9H492	2	others
DAPK2_HUMAN	Q9UIK4	7	kinase	ACBD5_HUMAN	Q5T8D3	1	others
DAPK3_HUMAN	O43293	4	kinase	CISD2_HUMAN	Q8N5K1	2	others
ABL2_HUMAN	P42684	7	kinase	NPC1_HUMAN	O15118	1	others
AAKB2_HUMAN	O43741	8	kinase	ZC12A_HUMAN	Q5D1E8	1	ion binding
AAPK2_HUMAN	P54646	5	kinase	MLP3C_HUMAN	Q9BXW4	2	others
PIM2_HUMAN	Q9P1W9	2	kinase	ATG13_HUMAN	O75143	1	others
AAKG1_HUMAN	P54619	3	kinase	WDFY3_HUMAN	Q8IZQ1	1	others
STK11_HUMAN	Q15831	1	kinase	GBRL2_HUMAN	P60520	1	protein binding
LRRK2_HUMAN	Q5S007	2	kinase	ATG12_HUMAN	O94817	2	others
PK3C3_HUMAN	Q8NEB9	5	kinase	A16L1_HUMAN	Q676U5	3	others
AAKB1_HUMAN	Q9Y478	1	kinase	ATG5_HUMAN	Q9H1Y0	3	others
TBK1_HUMAN	Q9UHD2	4	kinase	VPS51_HUMAN	Q9UID3	1	others
AAPK1_HUMAN	Q13131	1	kinase	FBX7_HUMAN	Q9Y3I1	1	others
ULK1_HUMAN	O75385	1	kinase	LYRIC_HUMAN	Q86UE4	1	transcription factor binding
ABL1_HUMAN	P00519	30	kinase	TCPR1_HUMAN	Q7Z6L1	1	others
MTOR_HUMAN	P42345	12	kinase	STX17_HUMAN	P56962	1	others
GBRAP_HUMAN	O95166	7	receptor	VAMP8_HUMAN	Q9BV40	1	others
OPTN_HUMAN	Q96CV9	3	receptor	SNP29_HUMAN	O95721	1	others

### Target prediction and pathways for autophagy-activating or autophagy-inhibiting compounds

**Figure 2:**
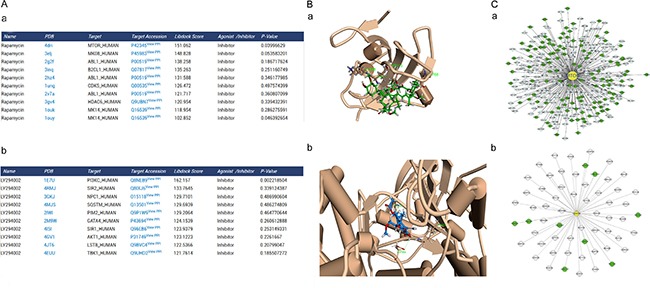
Predicted autophagic targets and related pathways from ACTP result page (**A**) The output pages for (a) rapamycin (CAS number: 53123–88–9) and (b) LY294002 (CAS number: 154447–36–6) were displayed. The dock scoring table displayed on the page shows the top 10 possible targets according to the dock score. (**B**) Snapshots of (a) rapamycin docked with mTOR and (b) LY294002 docked with PI3K (the highest scored target in the result table) were also shown. (**C**) Users can also see the target PPI network graphically by clicking the view PPI hyperlink in the superscript of the target Uniprot AC, (a) mTOR, (b) PI3K. The PPI network is displayed by the cytoscape web plugin.

The docking results were shown in a table of target proteins and include the top 10 docking scores and the *P*-value of the score. In this study, we used rapamycin and LY294002 as an example. We found that mTOR has the best binding score with rapamycin, 151.062; while PI3K has the best binding score with LY294002, 162.157 (Figure 2A). Rapamycin and LY294002 bound perfectly in the mTOR and PI3K inhibitor pocket, respectively. Moreover both of them had a similar conformation in different docking algorithms (Figure 2B).

To construct the global human PPI network based on PrePPI, we collected 24,035 human protein accession numbers from Uniprot and saved them in a text file. The results page was created using PHP with accession numbers from the text file and request interaction data. All the information were imported into MySQL database. As a result, 1.1 million PPIs were collected to construct the global network. We generated the ARP subnetwork and created the autophagy subnetwork, which contains 93,532 PPIs. The autophagy subnetwork data were also available on the “download” page. It was combined with PHP and MySQL web2.0 to generate a dynamic graphical network. For example, mTOR has 308 PPIs and PI3K has 60 PPIs in their cytoscape visual network. The target was in the center and was marked in yellow. The targets of high and low credible level ARP were displayed in green and gray, respectively (Figure 2C). Moreover we carried out an additional blind-test with 15 compounds with known targets (Supplementary Table S4). And, the result showed the predict targets with the significant Libdock score of 14 compounds contained the real target of the compound (10 compounds' real target had the top Libdock score). Only one compound's predicted targets result did not contain the real target. Thus, it suggests that ACTP has a reliable accuracy for predict the target of autophagy-activating or autophagy-inhibiting compounds.

### Webserver development

**Figure 3:**
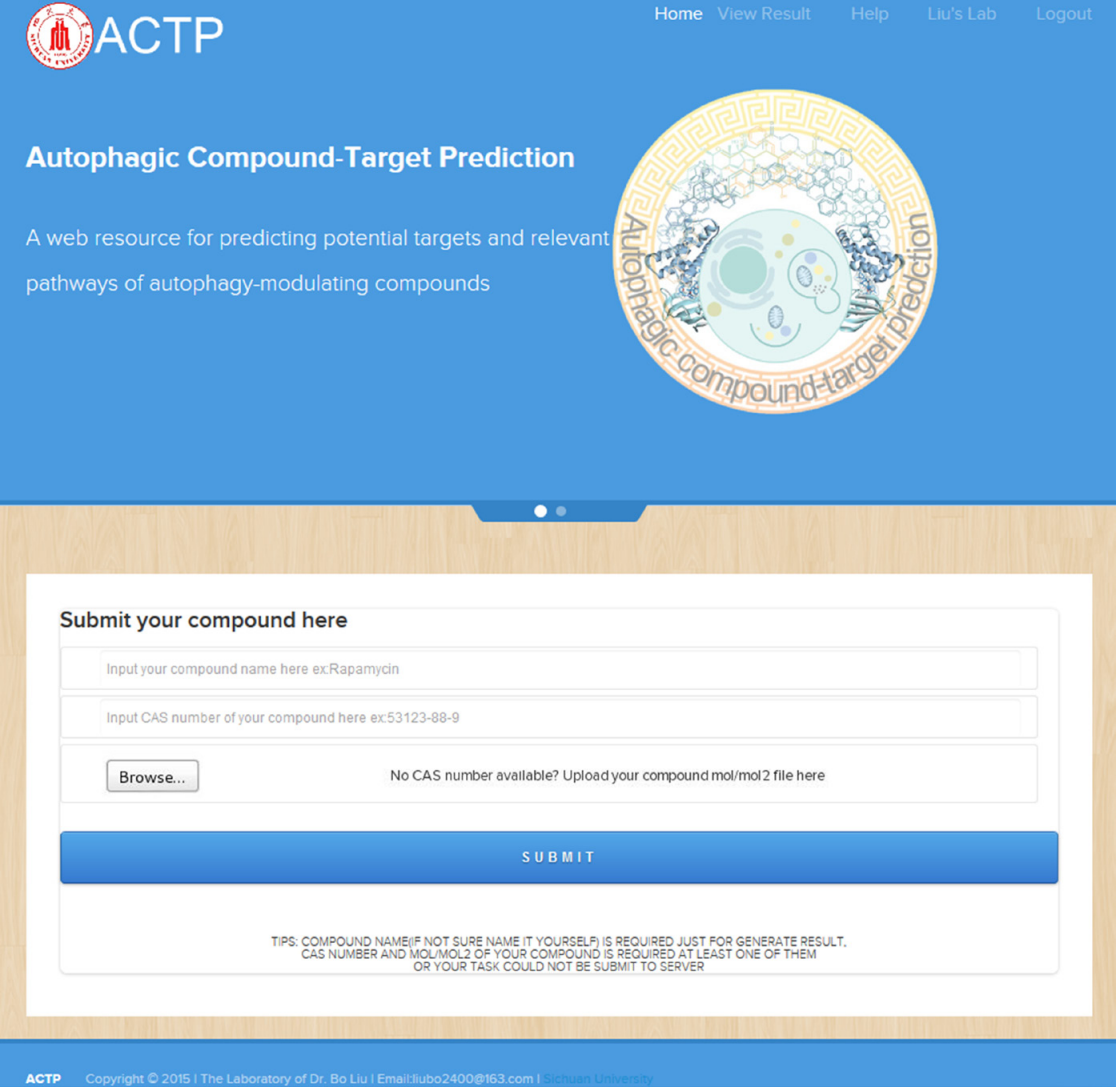
The ACTP user interface The simple user interface enables task submitting by inputting the compound name, CAS number, or by uploading a mol/mol2 formatted file. The pre-input example and tips help users become accustomed to the input format.

Based upon the above-mentioned results, we developed the ACTP webserver to offer a simple interface for users to submit compounds and predict their potential targets. For the first time, a user should create an account to submit compounds and view the results though the user interface. We support either CAS number or mol/mol2 files as the submission format. When submitted, the compound is sent to the Discovery Studio to perform virtual screening with ARPs. We would notify the users when the job is complete. If a user has submitted any compound previously, the webserver will display the results directly. The results page includes not only the docking scores and a snap-shot, but important information about the target proteins. For example, if rapamycin was submitted, the input can be either 53123–88–9 or a mol/mol2 file. Then, the task and process stage are shown on a user dashboard. When the task is complete, the user can click “VIEW” to see the score table, target information and PPIs (Figure 3). Currently, a due to the limitation of server is that a user could only submit 5 tasks per day.

## DISCUSSION

Autophagy may possess the contradictory functions because in addition to being primarily a survival mechanism, it can also lead to type II programmed cell death (type II PCD) under certain conditions [18, 19]. Our understanding of the relationship between autophagy and diseases has benefited from the availability of rapamycin and other autophagy-activating or autophagy-inhibiting agents, such as tamoxifen, chloroquine and resveratrol, which have been approved for potential clinical use [20, 21]. Several small-molecule compounds have been reported to activate or inhibit autophagy in different diseases. However, few of them has been purposefully designed as autophagic activators or inhibitors. Thus, it is urgent to find an avenue for rapidly screening and identifying a wealth of possible autophagy-activating or autophagy-inhibiting compounds without labor-intensive experiments.

Herein, we designed the Autophagic Compound-Target Prediction (ACTP) (http://actp.liu-lab.com/) webserver, which can predict a specific compound's autophagic targets and relevant pathways. We used a series of bioinformatics methods to assemble together for solving only one problem. When a given compound has been submitted, we could correspondingly predict its potential autophagic targets and relevant pathways for therapeutic purposes. There are some key points for our methods to construct the ACTP webserver. Firstly, the autophagy-related protein (ARP) data were collected and classified into different subclasses for accurate target identification. Secondly, autophagic targets and their relevant pathways were provided for possible mechanism analysis. Lastly but most importantly, autophagic targets and relevant pathways could be predicted according to given compounds by structure-based docking technique. Interestingly, the ACTP could provide a clue for themselves prone to activators or inhibitors of these predicted autophagic targets.

Of course, there are some limitations for ACTP. The binding sites of the reviewed targets are directly imported from PDB files; thus, ACTP cannot predict the binding of compounds to other pockets. Moreover, for many proteins, the structures are not available yet, and the homology modeling is not sufficiently accurate for prediction. Therefore, ACTP cannot currently confirm the results for these proteins. However, with a growing number of protein structures to be analyzed, we will continue to add some new protein structures, which could be used for accurate target prediction. Moreover, we plan to update the latest data every two months, enabling continuous improvement of the webserver and processes.

In summary, Autophagic Compound-Target Prediction (ACTP) may provide a basis for the rapid prediction of potential targets and relevant pathways for a given autophagy-modulating compound. These results will help a user to assess whether the submitted compound can activate or inhibit autophagy by targeting which kind of key autophagic proteins and also has a therapeutic potential on diseases. Importantly, ACTP will also provide a clue to guide further experimental validation on one or more autophagy-activating or autophagy-inhibiting compounds for future drug discovery.

## MATERIALS AND METHODS

### Target protein information collection and preprocessing

Autophagy-related proteins (ARPs) included genes or proteins that are associated with the Gene Ontology (GO) term “autophagy” (http://www.geneontology.org) [22]. The useful information on ARPs was extracted from Uniprot database (http://www.uniprot.org). Autophagic targets were classified based on their molecular functions. Targets were assigned to 9 functional target groups. Cluster analysis was deemed to be relevant if the over-represented functional groups contained at least 5 targets. Moreover, functional clustering was performed by the DAVID functional annotation tool (http://david.abcc.ncifcrf.gov/). The functional categories were GO terms that is related to molecular function (MF). Specific docking strategies were employed for different groups. For instance, kinase binding pockets were focused on the active sites, while antigens were focused on their interaction surfaces with other proteins. It may reduce the number of false positive results in *in silico* analysis [23, 24]. Also, the active sites were divided into two groups by their position for predicting if a compound is an inhibitor or agonist of the target [25, 26]. Taken a kinase as an example, inhibitors targeting active sites for kinases, the agonists were chose screening sites for according to the different regulation mechanism of kinases. For example, the AMPK agonist named compound 991 is envisaged to strengthen the interaction between the kinase and carbohydrate-binding module (CBM) to protect a major proportion of the active enzyme against dephosphorylation [25]. If available, ARP crystal structures were downloaded from the Protein Data Bank (PDB) website (www.rcsb.org) [27]. For proteins that have more than one PDB entry, we screened the PDB files by resolution and sequence length until only one PDB entry remained. For proteins without crystal structure, we created homology modeling from sequences using Discovery Studio 3.5 (Accelrys, San Diego, California, United States). Sequence data were downloaded from Uniprot in FASTA format, and the templates were identified using BLASTP (Basic Local Alignment Search Tool) (http://blast.ncbi.nlm.nih.gov). ARPs were divided into two credibility levels (high and low) according to their review status in Uniprot.

### Protein-protein interaction (PPI) network construction

The cellular biological processes of specific targets were predicted based on the global architecture of PPI network. We used an in-house PHP script to construct Autophagy interaction networks (AINs) based on the global PPI network were from PrePPI database (https://bhapp.c2b2.columbia.edu/PrePPI) [28] and Uniprot accession numbers. The ARP accession numbers were used to generate an AIN subnetwork. PPIs with different credible levels were marked in ACTP. The interactions were recorded in SQL format, which could be imported into MySQL database. The Cytoscape web plug-in was used to visualize the interactions [29].

### Webserver generation

The ACTP webserver was generated with Linux, Apache, MySQL and PHP. Users can inquiry the database with their private data through the web interface. Currently, all major web browsers are supported. The processed results will be returned to the website. Web 2.0 technologies (i.e., JavaScript/AJAX and CSS functionalities) enables interactive data analysis. For example, based on AJAX and flash, ARP interaction networks can be indexed by accession numbers and visualized on the web page with Cytoscape web.

### Reverse docking

Reverse docking is the virtual screening of targets by given compounds based on various scoring functions. Reverse docking allows a user to find the protein targets which can bind to a particular ligand [30]. We performed reverse docking with Libdock protocol [31], which is a high-throughput docking algorithm that positions catalyst-generated compound conformations in protein hotspots. Before docking, force fields including energies and forces on each particle in a system were applied with CHARMM [32] to define the positional relationships among atoms and to detect their energy. The binding site image consists of a list of non-polar hot spots, and positions in the binding site that were favorable for a non-polar atom to bind. Polar hot spot positions in the binding site were favorable for the binding of a hydrogen bond donor or acceptor. For Libdock algorithm, a given ligand conformation was put into the binding site as a rigid body and the atoms of the ligand were matched to the appropriate hot spots. The conformations were ranked using the following score:

Score=Strain−0.1XSASA

where SASA is the solvent accessible surface area of a particular conformation measured in Å^2^ and the strain is in units of kcal/mol. A match then determines the unique rigid body transformation that minimizes the following equation:

$$\begin{array}{ccc} H_{j}-RA_{j} & - & T\vee^{2}\\ I(R,T) & = & \sum_{j=1}^{3} \end{array}$$

where R is a 3 × 3 rotation matrix and T is a translation vector. A single conformation can produce up to 10,000 matches. Thus, in the final stage, the matches were clustered after ranking, and only the top 25–100 entries were chosen for the next stage. Two values were reported as the measures of success of the two scores in pulling out active compounds. The first step of these measurements is the enrichment factor and is given by the following equation:

$$\text{Enrichment}=\frac{a/n}{A/N}$$

N is the number of compounds in the library; A is the number of active compounds; and a is the number of active compounds in the top n compounds. The second value is the statistical significance of the enrichment and is given by the following equation:

$$\text{Significance}=\sum_{k=a}^{A}\frac{\left(\begin{array}{c} A\\ K \end{array}\right)\left(\begin{array}{c} N-A\\ n-k \end{array}\right)}{\left(\begin{array}{c} N\\ n \end{array}\right)}$$

N, A, n, and a are defined as the enrichment. Comparing with other dock algorithm Libdock is quicker and support concurrent computation as well. Moreover protocol in Discovery Studio can be used to perform the Libdock algorithm for a series of proteins. Thus, Libdock is a suitable algorithm for high throughput identifying the various conformations of compounds within a receptor. Target-compound interactions were further optimized by molecular dynamics using CHARMM and Clean Geometry function of Discovery Studio. A *T*-test was also add to analyze the significance of the Libdock score of each target.

## SUPPLEMENTARY MATERIALS TABLES










