# Supplementary material for: ACTP: A webserver for predicting potential targets and relevant pathways of autophagy-modulating compounds
Source: Oncotarget. 2016 Jan 25;7(9):10015–22. doi: 10.18632/oncotarget.7015 (PMC4891100; doi:10.18632/oncotarget.7015)
Supplement: Supplementary file 1 [file oncotarget-07-10015-s001.pdf]

# ACTP: A webserver for predicting potential targets and relevant pathways of autophagy-modulating compounds

## Supplementary Materials

**Supplementary Table S1: The detailed information of reviewed autophagic protein targets based on crystal structures**

**Supplementary Table S2: Reviewed autophagic protein targets based on their sequences**

**Supplementary Table S3: Unreviewed autophagic protein targets**

**Supplementary Table S4: Additional blind-test results for 15 compounds with known targets**
